# Supplementary material for: Healthcare utilization, mortality, and cardiovascular events following GLP1-RA initiation in chronic kidney disease
Source: Nat Commun. 2024 Dec 5;15:10623. doi: 10.1038/s41467-024-54009-3 (PMC11621321; doi:10.1038/s41467-024-54009-3)
Supplement: Supplementary file 1 — Supplementary Information [file 41467_2024_54009_MOESM1_ESM.pdf]

Supplementary online material

Healthcare Utilization, Mortality, and Cardiovascular Events following GLP1-RA initiation In Chronic Kidney Disease

Shuyao Zhang, Fnu Sidra, Carlos Alvarez, Mustafa Kinaan, Ildiko Lingvay, Ishak Mansi

Supplementary Figures: .... Page 2

- Figure S1 – Study Intervals
- Figure S2 - Overlap weighting of propensity score distribution between the groups (A) before matching, and (B) after propensity score matching.
- Figure S3 – Forest Plot of subgroup analysis
- Figure S4 - Kaplan-Meier curves using per-protocol analysis

Supplementary Tables: .... Page 9

- Table S1 - Incidence and progression of albuminuria during follow up period
- Table S2 - Table of primary and secondary outcomes of overall cohort, excluding ESRD
- Table S3 - Table of primary and secondary outcomes of the propensity-matched cohort, adjusted for A1c and other hypoglycemic agents at time of follow-up
- Table S4 – Table of post-hoc safety outcomes
- Table S5 – Table of results of per-protocol analysis
- Table S6 - Table of drugs included in study with proportions
- Table S7 – Summary of the protocol
- Table S8- Definition of administrative codes used in the study outcomes
- Table S9 - Administrative codes used in definitions of baseline characteristics
- Table S10 – LOINC codes used to identify laboratory tests
- Table S11 – Table of missing value

SUPPLEMENTARY FIGURES

<u>Supplemental Figure S1: Study intervals (intention-to-treat primary analysis):</u> Schematic of study intervals and intention-to-treat protocol. Prior to propensity score matching, the GLP-1RA treatment arm consisted of 26,997 patients, of which 8,315 (30.8%) discontinued medication after a mean (SD) period of 434 (490) days. Of the patients that discontinued, 819 (2.2%) later started DPP4i. The DPP4i treatment arm consisted of 37,708, of which 17,658 (46.8%) discontinued treatment after a mean (SD) period of 603 (634) days. Of the patients that discontinued DPP4i, 6,727 (24.9%) were later started on GLP-1RA (these patients were censored at date of GLP-1RA initiation).

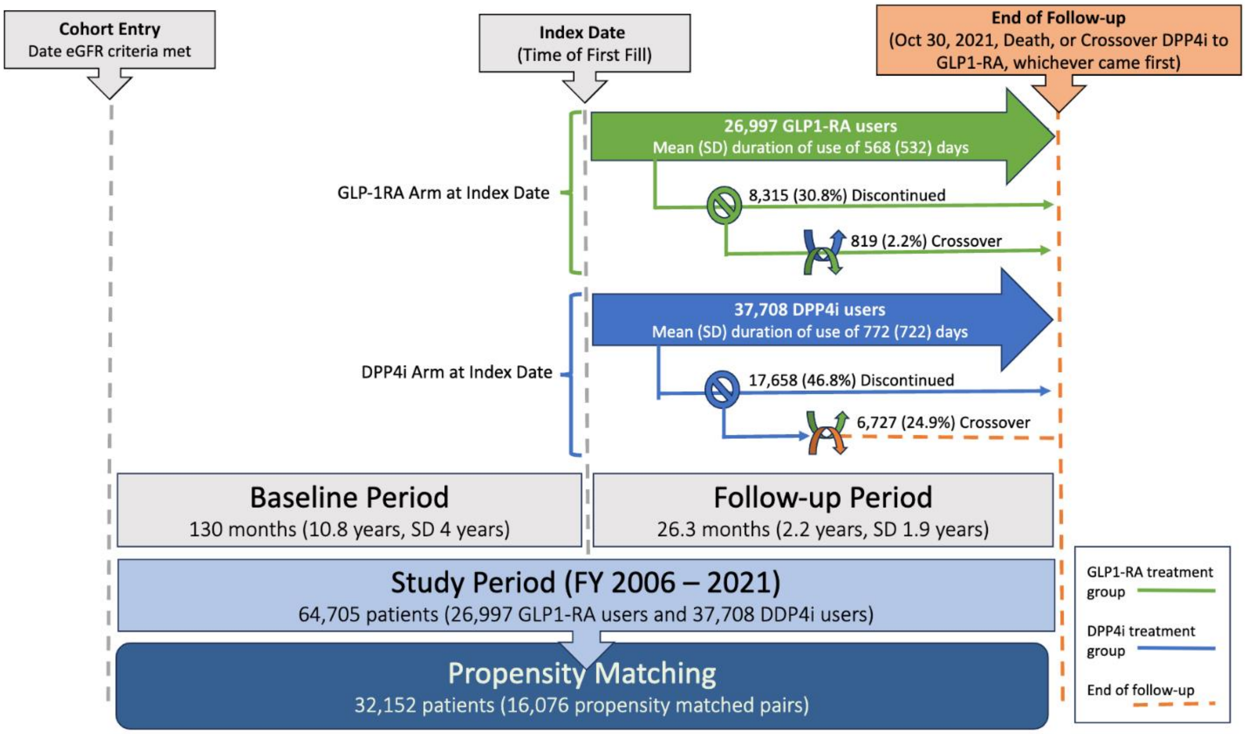

Figure Legend: GLP1-RA, glucagon like peptide 1 receptor agonists; DPP4i, dipeptidyl peptidase 4.

Study period = FY 2006-2021. Cohort entry = the date the above eGFR criteria was met. Index date = date of first fill of GLP1RA or DPP4i. Baseline period = first available encounter to index date. Follow-up period = index date to the end of study (Oct 30, 2021), death, or switch to GLP1-RA in the DDP4i group – whichever came first.

Supplemental Figure S2: Kernel graphs of propensity score before (Figure S2-A) and after (Figure S2-B) matching.

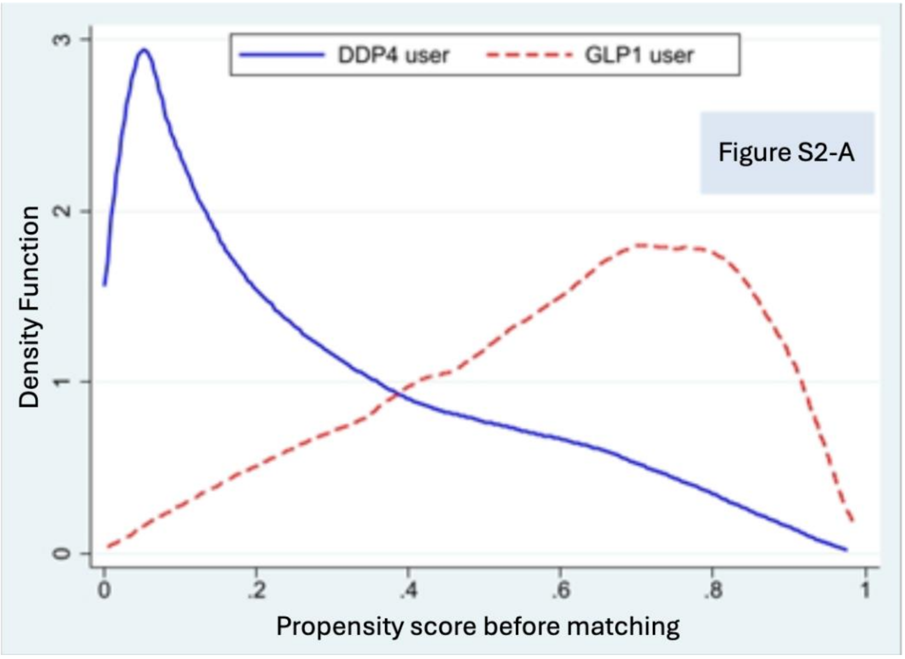

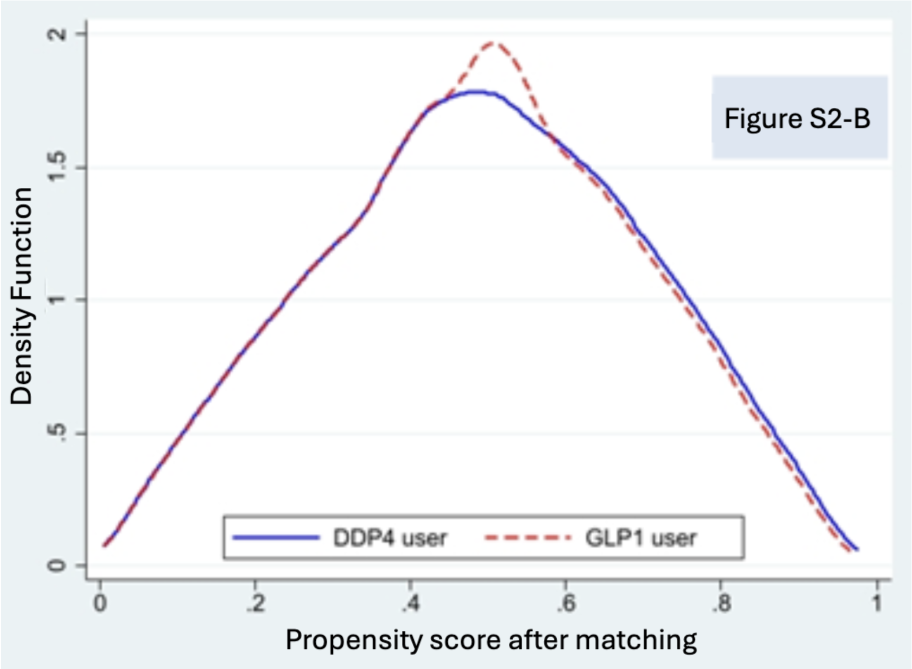

Figure Legend: GLP1, glucagon like peptide 1; DPP4i, dipeptidyl peptidase 4 inhibitors; HR

Supplemental Figure S3: Forest plot diagram illustration coefficient of regression or adjusted odds ratio by subgroup for the following outcomes: acute healthcare utilization (panel A), CV events (panel B), CKD progression (panel C), and All-cause death (panel D).

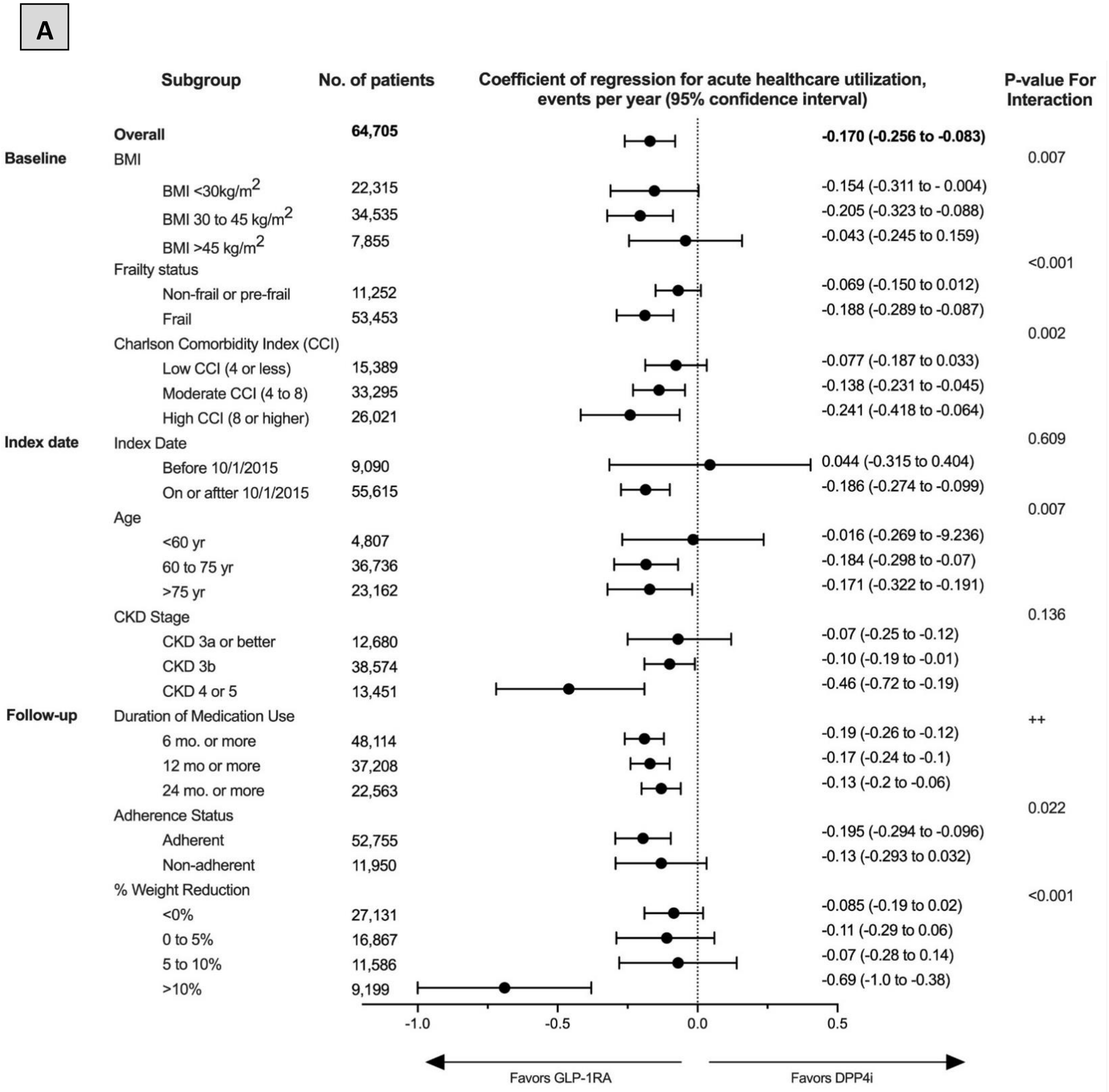

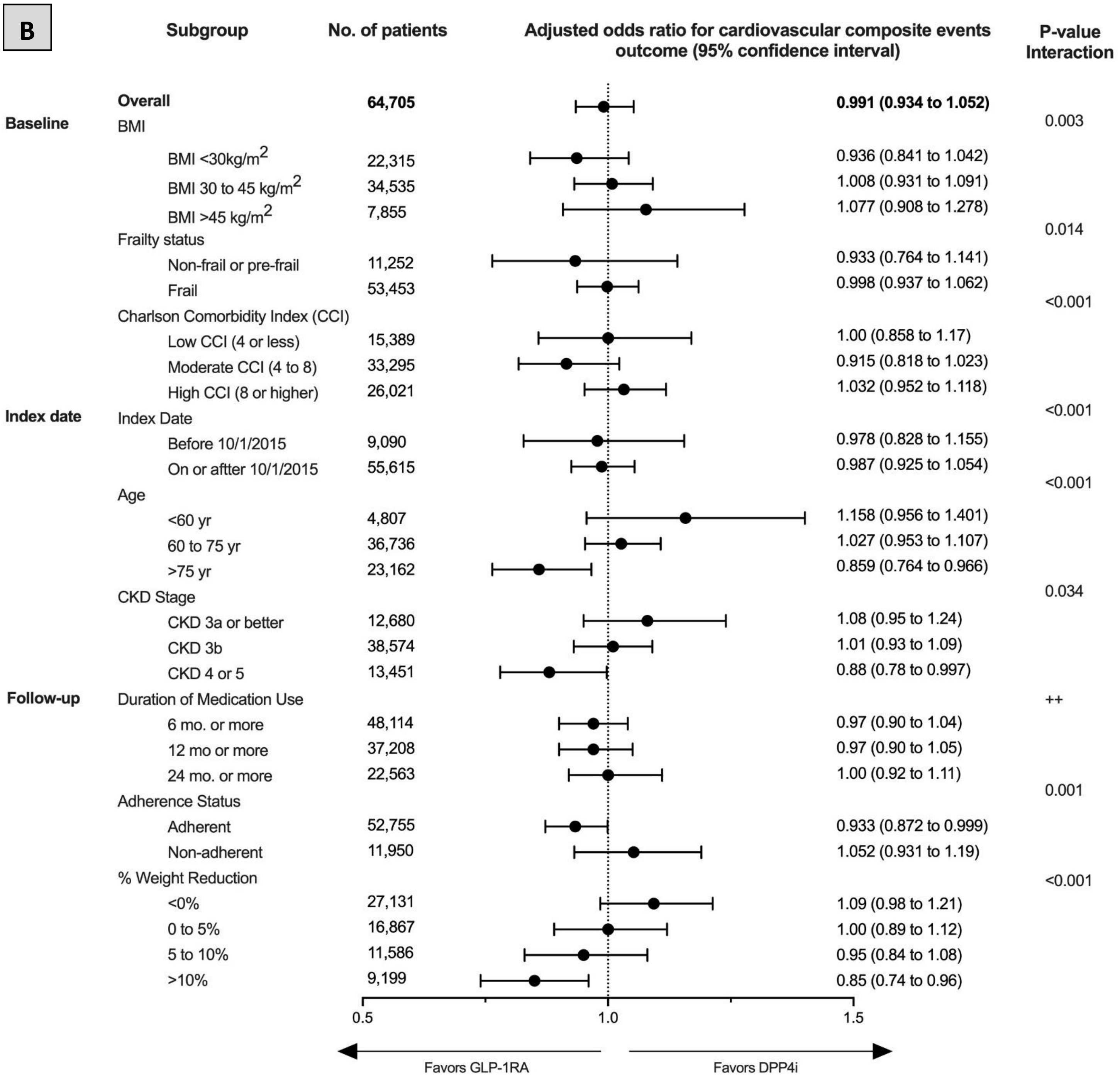
B
Subgroup
No. of patients
Adjusted odds ratio for cardiovascular composite events outcome (95% confidence interval)
P-value Interaction
Overall
64,705
0.991 (0.934 to 1.052)
Baseline
BMI
0.003
BMI <30kg/m2
22,315
0.936 (0.841 to 1.042)
BMI 30 to 45 kg/m2
34,535
1.008 (0.931 to 1.091)
BMI >45 kg/m2
7,855
1.077 (0.908 to 1.278)
Frailty status
0.014
Non-frail or pre-frail
11,252
0.933 (0.764 to 1.141)
Frail
53,453
0.998 (0.937 to 1.062)
Charlson Comorbidity Index (CCI)
<0.001
Low CCI (4 or less)
15,389
1.00 (0.858 to 1.17)
Moderate CCI (4 to 8)
33,295
0.915 (0.818 to 1.023)
High CCI (8 or higher)
26,021
1.032 (0.952 to 1.118)
Index date
Index Date
<0.001
Before 10/1/2015
9,090
0.978 (0.828 to 1.155)
On or aftter 10/1/2015
55,615
0.987 (0.925 to 1.054)
Age
<0.001
<60 yr
4,807
1.158 (0.956 to 1.401)
60 to 75 yr
36,736
1.027 (0.953 to 1.107)
>75 yr
23,162
0.859 (0.764 to 0.966)
CKD Stage
0.034
CKD 3a or better
12,680
1.08 (0.95 to 1.24)
CKD 3b
38,574
1.01 (0.93 to 1.09)
CKD 4 or 5
13,451
0.88 (0.78 to 0.997)
Follow-up
Duration of Medication Use
++
6 mo. or more
48,114
0.97 (0.90 to 1.04)
12 mo or more
37,208
0.97 (0.90 to 1.05)
24 mo. or more
22,563
1.00 (0.92 to 1.11)
Adherence Status
0.001
Adherent
52,755
0.933 (0.872 to 0.999)
Non-adherent
11,950
1.052 (0.931 to 1.19)
% Weight Reduction
<0.001
<0%
27,131
1.09 (0.98 to 1.21)
0 to 5%
16,867
1.00 (0.89 to 1.12)
5 to 10%
11,586
0.95 (0.84 to 1.08)
>10%
9,199
0.85 (0.74 to 0.96)
0.5
1.0
1.5
Favors GLP-1RA
Favors DPP4i

C

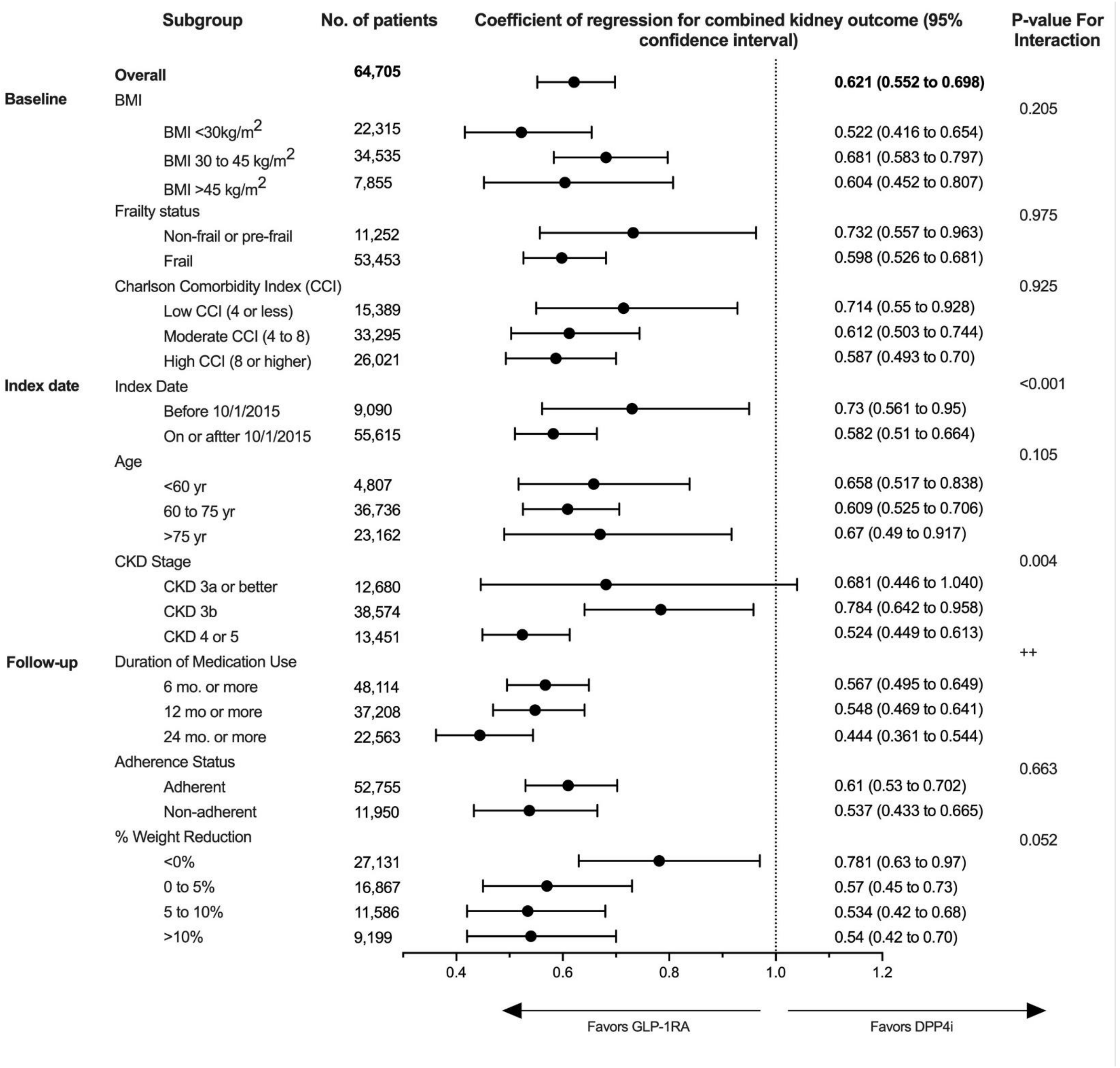
Subgroup
No. of patients
Coefficient of regression for combined kidney outcome (95% confidence interval)
P-value For Interaction
Overall 64,705 0.621 (0.552 to 0.698)
Baseline
BMI 0.205
BMI <30kg/m2 22,315 0.522 (0.416 to 0.654)
BMI 30 to 45 kg/m2 34,535 0.681 (0.583 to 0.797)
BMI >45 kg/m2 7,855 0.604 (0.452 to 0.807)
Frailty status 0.975
Non-frail or pre-frail 11,252 0.732 (0.557 to 0.963)
Frail 53,453 0.598 (0.526 to 0.681)
Charlson Comorbidity Index (CCI) 0.925
Low CCI (4 or less) 15,389 0.714 (0.55 to 0.928)
Moderate CCI (4 to 8) 33,295 0.612 (0.503 to 0.744)
High CCI (8 or higher) 26,021 0.587 (0.493 to 0.70)
Index date
Index Date <0.001
Before 10/1/2015 9,090 0.73 (0.561 to 0.95)
On or aftter 10/1/2015 55,615 0.582 (0.51 to 0.664)
Age 0.105
<60 yr 4,807 0.658 (0.517 to 0.838)
60 to 75 yr 36,736 0.609 (0.525 to 0.706)
>75 yr 23,162 0.67 (0.49 to 0.917)
CKD Stage 0.004
CKD 3a or better 12,680 0.681 (0.446 to 1.040)
CKD 3b 38,574 0.784 (0.642 to 0.958)
CKD 4 or 5 13,451 0.524 (0.449 to 0.613)
Follow-up
Duration of Medication Use ++
6 mo. or more 48,114 0.567 (0.495 to 0.649)
12 mo or more 37,208 0.548 (0.469 to 0.641)
24 mo. or more 22,563 0.444 (0.361 to 0.544)
Adherence Status 0.663
Adherent 52,755 0.61 (0.53 to 0.702)
Non-adherent 11,950 0.537 (0.433 to 0.665)
% Weight Reduction 0.052
<0% 27,131 0.781 (0.63 to 0.97)
0 to 5% 16,867 0.57 (0.45 to 0.73)
5 to 10% 11,586 0.534 (0.42 to 0.68)
>10% 9,199 0.54 (0.42 to 0.70)
0.4
0.6
0.8
1.0
1.2
Favors GLP-1RA
Favors DPP4i

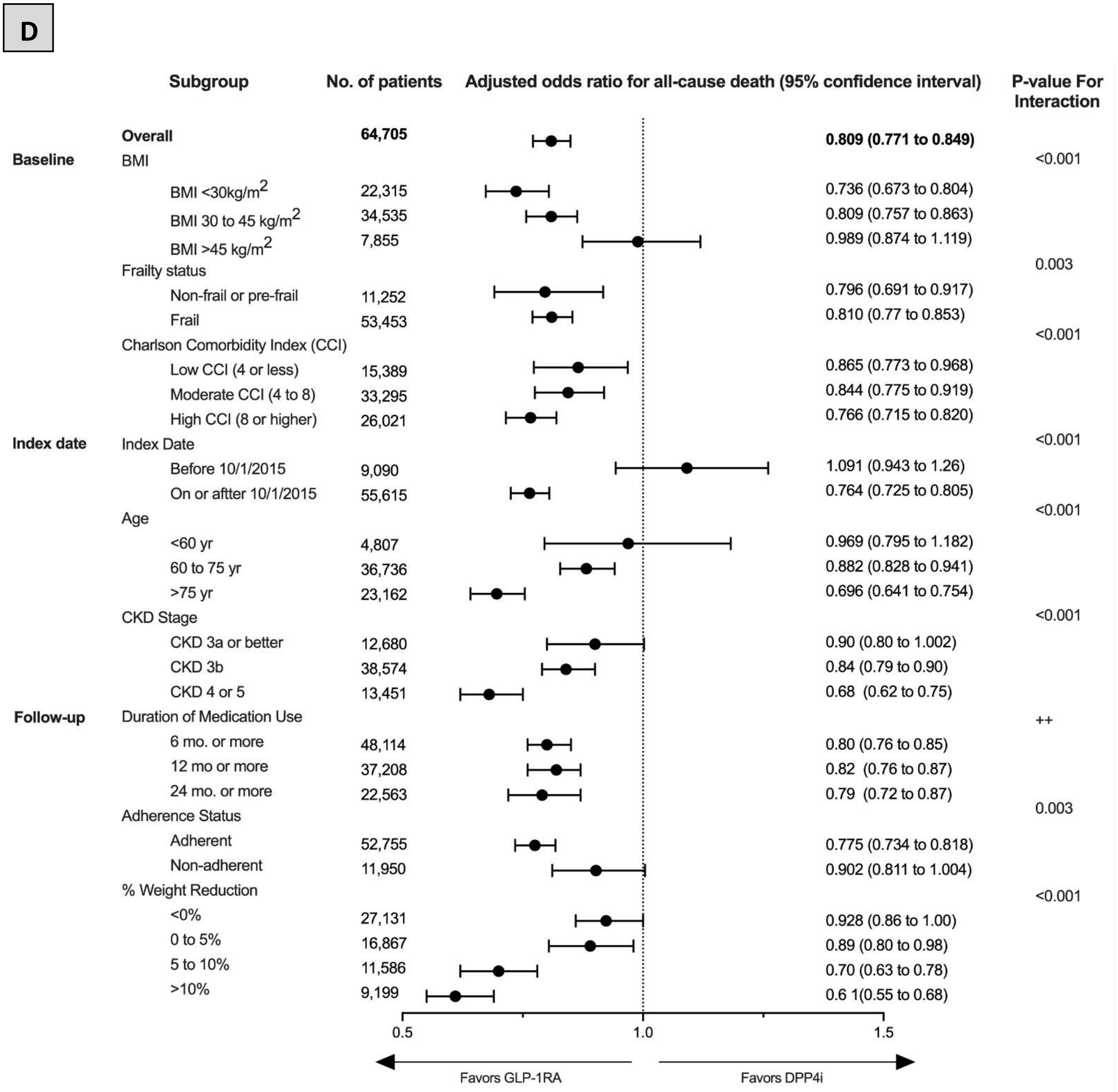

Figure Legend: Analyses are adjusted for propensity score. Applying Bonferroni correction for multiple comparisons, p<0.0012 indicates statistically significant interaction.
++p-value for interaction not calculated due to overlap among subgroups
BMI, body mass index; CKD, chronic kidney disease; yr, year; mo. Months
% weight reduction expressed as [weight at follow-up – weight at index date] / [weight at index date].

Supplemental Figure S4: Kaplan-Meier curves for time to first composite cardiovascular event using per-protocol analysis and total mortality.

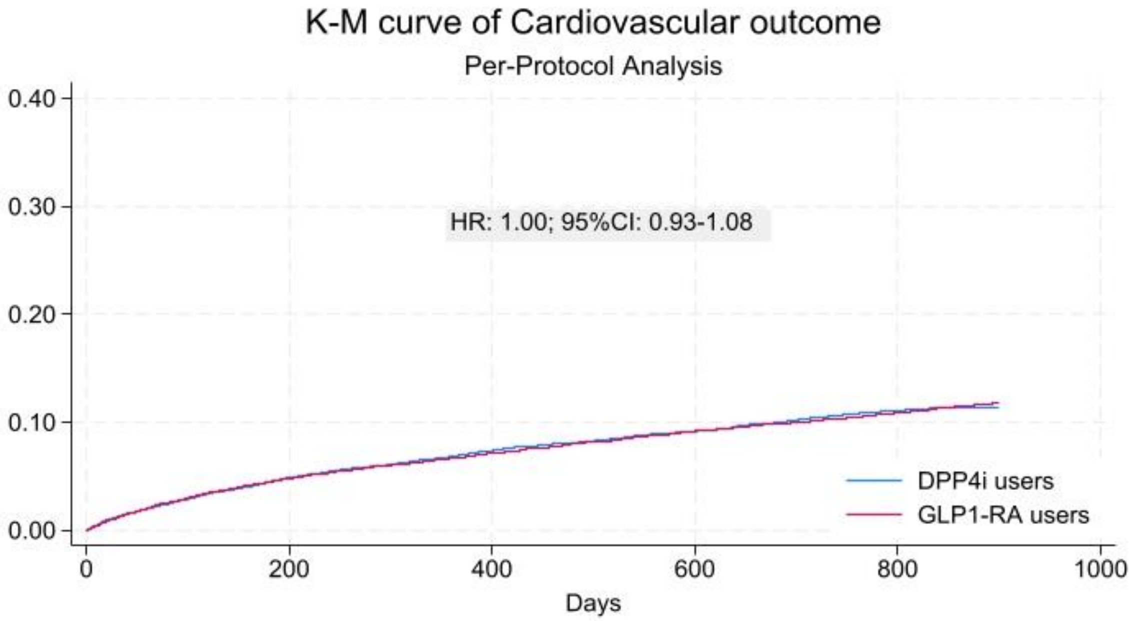

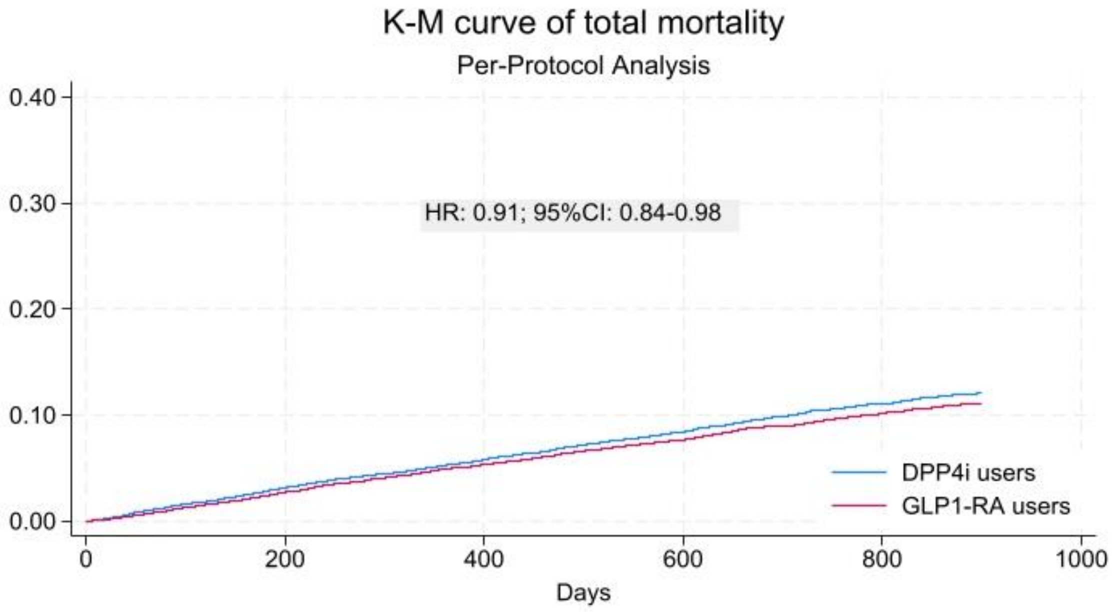

Figure Legend: K-M, Kaplan Meier; GLP1-RA, glucagon like peptide 1 receptor agonists; DPP4i, dipeptidyl peptidase 4 inhibitors; HR, hazard ratio; CI, confidence interval.

SUPPLEMENTARY TABLES

<u>Supplemental Table S1:</u>

A) Table S1-A: Incidence of albuminuria during baseline period.
B) Table S1-B: Incidence and progression of albuminuria during follow up period.

**Table S1-A. Incidence of albuminuria during baseline period**			
	**GLP1-RA**	**DPP4i**	**Standardized Difference**
	**n= 16,076**	**n= 16,076**	
Mean albumin in urine in mg			-0.185
Missing	1,462 (9.09%)	1,601 (9.96%)	
<30	8,802 (54.75%)	9,120 (56.73%)	
>= 3 to <300	4,680 (29.11%)	4,792 (29.81%)	
>=300 to <3000	793 (4.93%)	869 (5.41%)	
> 3000	21 (0.13%)	12 (0.07%)	

**Table S1-B. Incidence and progression of albuminuria during follow up period**				
	**GLP1-RA**	**DPP4i**	**Odds ratio (95%CI)***	**p-value**
	**n= 16,076**	**n= 16,076**		
Mean albumin level in urine in mg				
Missing	6,398 (39.80%)	6,900 (42.92%)	-	
<30	5,480 (34.09%)	5,029 (31.28%)	-	
>= 30 to <300	3,415 (21.24%)	3,293 (20.48%)	-	
>=300 to <3000	759 (4.72%)	839 (5.220%)	-	
> 3000	24 (0.15%)	15 (0.09%)		<0.001
Progression of albuminuria during follow up period	1,662 (10.34%)	1,737 (10.80%)	0.95 (0.89 - 1.02)	0.17

Regression of albuminuria during follow up period	809 (5.03%)	742 (4.62%)	1.10 (0.99-1.12)	0.08

Progression of albuminuria was defined as stepwise categorical increase in albuminuria (<30; >= 30 to <300; >=300 to <3000; or > 3000) compared to mean albuminuria at baseline.

Regression of albuminuria was defined as stepwise categorical decrease in albuminuria (<30; >= 3 to <300; >=300 to <3000; or > 3000) compared to mean albuminuria at baseline.

*adjusting for propensity score

Supplemental Table S2: Primary and Secondary Outcomes in overall cohort excluding subjects with eGFR <15 mL per min per 1.73 m2.

Table S2. Primary and Secondary Outcomes in overall cohort excluding subjects with eGFR <15 mL per min per 1.73 m2

	**GLP1-RA**	**DPP4i**	**coefficient of regression or odds ratio (95%CI)***	**p-value**
	**n= 26,247**	**n= 36,205**		
Rate of acute healthcare utilization, EPY	1.49 (4.41)	1.53 (4.40)	-0.178 (-0.26 to -0.09) [a]	<0.001
Cardiovascular composite event outcome	2813 (10.72%)	4,312 (11.91)	0.997 (0.94 - 1.06)[b]	0.913
Composite outcome of CKD progression	578 (2.2%)	1291 (3.57%)	0.61 (0.54 - 0.69)[b]	<0.001
Death at follow-up	3,936 (15%)	10,927 (30.18%)	0.824 (0.78 - 0.87)[b]	<0.001

*Multivariate linear regression analysis was performed for annual rate of healthcare utilization. Logistic regression analysis performed for all other outcomes, adjusting for propensity score. Each outcome was assessed in a separate model where the outcome was the dependent variable and use of GLP1-RA or DPP4i as predictor variable.

Data are mean (SD) or numbers (%) unless stated otherwise. [a]Coefficient of regression; [b]Odds ratio; EPY, event per patient per year; GLP1-RA, glucagon like peptide 1 receptor agonists; DPP4i, dipeptidyl peptidase 4 inhibitors.

Supplemental Table S3:

Primary and secondary outcomes of the PS-matched cohort, adjusting for A1c and use of other hypoglycemic agents at time of follow-up. Average A1c at time of follow-up was 7.915 (1.247) and 7.742 (1.267) within the GLP-1RA and DPP4i groups respectively; standardized difference= -0.137. Classes of anti-hyperglycemic agents included glitazones, glinides, insulins, metformin, SGLT2i, and sulfonylureas.

**Primary and Secondary Outcomes in PS-matched cohort (adjusting for A1c and use of other hypoglycemic agents during follow-up)**				
	**GLP1-RA**	**DPP4i**	**coefficient of regression or odds ratio (95%CI)***	**p-value**
	**n= 16,076**	**n= 16,076**		
Rate of acute healthcare utilization, EPY	1.524 (4.828)	1.672 (4.56)	-0.162 (-0.261 to -0.063)[a]	0.001
Cardiovascular composite event outcome	1757 (10.93%)	1782 (11.08%)	0.974 (0.906 to 1.047)[b]	0.47
Composite outcome of CKD progression	359 (2.23%)	557 (3.46%)	0.618 (0.537 to 0.71)[b]	<0.001
Death at follow-up	2847 (17.71%)	3287 (20.45%)	0.867 (0.825 to 0.922)[b]	<0.001

*Multivariate linear regression analysis was performed for annual rate of healthcare utilization. Logistic regression analysis performed for all other outcomes, adjusting for propensity score. Each outcome was assessed in a separate model where the outcome was the dependent variable and use of GLP1-RA or DPP4i as predictor variable.

Data are mean (SD) or numbers (%) unless stated otherwise. [a]Coefficient of regression; [b]Odds ratio; EPY, event per patient per year; GLP1-RA, glucagon like peptide 1 receptor agonists; DPP4i, dipeptidyl peptidase 4 inhibitors.

Supplemental Table S4: Safety outcomes in the propensity-score matched cohort.

Supplemental Table S4. Safety Outcomes in PS-matched cohort				
	**GLP1-RA**	**DPP4i**	**Odds ratio (95%CI)**	**p-value**
	**n= 16,076**	**n= 16,076**		
Hypoglycemic events	3743 (23.3%)	3644 (22.7%)	1.13 (1.05-1.21)	0.001
Gastrointestinal symptoms	1657 (10.3%)	1487 (9.3%)	1.04 (0.98-1.09)	0.19

Data are numbers (%) unless stated otherwise. Each outcome was assessed in a separate model where the outcome was the dependent variable and use of GLP1-RA or DPP4i as predictor variable.

Supplemental Table S5: Outcomes in propensity-score matched cohort using per-protocol analysis.

Supplemental Table S5: Outcomes in PS-matched cohort using per-protocol analysis				
	**GLP1-RA n= 16,057**	**DPP4i n= 16,057**	**Parameter Estimate (95%CI)***	**p-value**
Rate of acute healthcare utilization, EPY: mean (SD)	1.53 (5.04)	1.70 (4.63)	B: -0.17 (-0.28 to -0.06)	0.002
Cardiovascular composite event outcome	1,342 (8.36%)	1,320 (8.36%)	OR: 1.02 (0.94 – 1.10)	0.66
Composite outcome of CKD progression	214 (1.33%)	347 (2.16%)	OR: 0.61 (0.52-0.73)	<0.001
Death at follow-up	1,302 (8.11%)	1,354 (8.43%)	OR: 0.96 (0.89-1.04)	0.29

*Multivariate linear regression analysis was performed for annual rate of healthcare utilization. Logistic regression analysis performed for all other outcomes, adjusting for propensity score. Each outcome was assessed in a separate model where the outcome was the dependent variable and use of GLP1-RA or DPP4i as predictor variable.

Data are mean (SD) or numbers (%) unless stated otherwise. B, Coefficient of regression; OR, Odds ratio; EPY, event per patient per year; GLP1-RA, glucagon like peptide 1 receptor agonists; DPP4i, dipeptidyl peptidase 4 inhibitors.es.

Supplemental Table S6: within each group (GLP1-RA vs DPP4i).

Table S6. Proportion of medications prescribed

**GLP1-RA**		**DDP4i**	
Medication	Proportions	Medication	Proportions
Albiglutide	0.03065	Alogliptin	0.34227
Dulaglutide	0.25651	Linagliptin	0.08744
Exenatide	0.06610	Saxagliptin	0.39293
Liraglutide	0.34486	Sitagliptin	0.17736
Lixisenatide	0.00015		
Semaglutide	0.30172		

GLP1-RA, glucagon like peptide 1 receptor agonists; DPP4i, dipeptidyl peptidase 4 inhibitors.

Supplemental Table S7: Summary of the protocol with emulation of a randomized control trial

Protocol Component	Hypothetical RCT	Emulation of RCT
Key Eligibility criteria	Adults aged 35 years who are regular VHA users with moderate to advanced CKD with two or more consecutive eGFR values <45 mL/min/1.73m² obtained over a span of three consecutive months.	Adults aged 35 years who are regular VHA users with moderate to advanced CKD with two or more consecutive eGFR values <45 mL/min/1.73m² obtained over a span of three consecutive months.
Treatment strategies	Placebo-controlled trial comparing placebo and GLP-1RA.	Active comparator using DPP4i instead of placebo to help select treatment groups similar in treatment indications, and use of new-user design to assess patients' pretreatment characteristics and capture events during follow-up period.
Assignment procedures/Randomization	Randomization - participants are randomly assigned to placebo or treatment.	Propensity-score matching on 120 baseline characteristics to emulate randomization. Patients are aware of the strategy to which they have been "assigned".
Follow-up period	Starts at randomization and ends at study end (October 30, 2021), death, or initiation of a GLP1-RA in the placebo group, whichever occurs first.	Starts at index date (date of GLP-1RA or DPP4i initiation) and ends at study end (October 30, 2021), death, or initiation of a GLP1-RA in the DPP4i group, whichever occurs first.
Primary Outcome	Annual rate of acute healthcare utilization.	Annual rate of acute healthcare utilization.
Secondary Outcomes	All cause-mortality, incidence of cardiovascular composite event outcome, and ad-hoc composite kidney outcome.	All cause-mortality, incidence of cardiovascular composite event outcome and ad-hoc composite kidney outcome.
Analysis plan	Intention-to treat.	Patients are analyzed regardless of medication discontinuation.
	Per-protocol analysis.	Follow up period ended, or events censored after discontinuation of the medication in their respective group

Supplemental Table S8: **Definitions of administrative codes (ICD-9 and ICD-10) used in the study outcomes.**

Table S8. Definitions of administrative codes (ICD-9 and ICD-10) used in the study outcomes

	**ICD-9**	**ICD-10**
**Acute myocardial infarction: These codes has a positive predictive value (PPV) of 0.91.**[1]	41000', '41001', '41002', '41010', '41011', '41012', '41020', '41021', '41022', '41030', '41031', '41032', '41040', '41041', '41042', '41050', '41051', '41052', '41060', '41061', '41062', '41070', '41071', '41072', '41080', '41081', '41082', '41090', '41091', '41092', '412'	I2101', 'I2102', 'I2109', 'I2111', 'I2119', 'I2121', 'I2129', 'I213', 'I214', 'I220', 'I221', 'I222', 'I228', 'I229', 'I252'
**Acute cerebrovascular disease: these codes have PPV 73 for <60 yr-old and PPV 82 for >60 yr old, NPV 1; specificity 100%.**[2]	430 431 436 434 434.0 434.00 434.01 434.1 434.10 434.11 434.9 434.90 434.91	I60, I61, I63, I64 I60 I60.0 I60.1 I60.2 I60.3 I60.4 I60.5 I60.6 I60.7 I60.8 I60.9 I61 I61.0 I61.1 I61.2 I61.3 I61.4 I61.5 I61.6 I61.8 I61.9 I63 I63.0 I63.1 I63.2 I63.3 I63.4 I63.5 I63.6 I63.8 I63.9 I64
**Cardiac arrest/ventricular fibrillation**	AHRQ-CCS category 107	I462' 'I468' 'I469' 'I4901' 'I4902' 'I97120' 'I97121' 'I97711' 'O0336' 'O0386' 'O0486' 'O0486' 'O0736' 'O0881' 'O29111' 'O29112' 'O29113' 'O29119' 'Z8674'
**Percutaneous coronary intervention procedure codes**[1]	0066', '1755', '3601', '3602', '3605', '3606', '3607', '3609'	0270046', '027004Z', '02700D6', '02700DZ', '02700T6', '02700TZ', '02700Z6', '02700ZZ', '0270346', '027034Z', '02703D6', '02703DZ', '02703T6', '02703TZ', '02703Z6', '02703ZZ', '0270446', '027044Z', '02704D6', '02704DZ', '02704T6', '02704TZ', '02704Z6', '02704ZZ', '0271046', '027104Z', '02710D6', '02710DZ', '02710T6', '02710TZ', '02710Z6', '02710ZZ', '0271346', '027134Z', '02713D6', '02713DZ', '02713T6', '02713TZ', '02713Z6', '02713ZZ', '0271446', '027144Z', '02714D6', '02714DZ', '02714T6', '02714TZ', '02714Z6', '02714ZZ', '0272046', '027204Z', '02720D6', '02720DZ', '02720T6', '02720TZ', '02720Z6', '02720ZZ', '0272346', '027234Z', '02723D6', '02723DZ',

		'02723T6', '02723TZ', '02723Z6', '02723ZZ', '0272446', '027244Z', '02724D6', '02724DZ', '02724T6', '02724TZ', '02724Z6', '02724ZZ', '0273046', '027304Z', '02730D6', '02730DZ', '02730T6', '02730TZ', '02730Z6', '02730ZZ', '0273346', '027334Z', '02733D6', '02733DZ', '02733T6', '02733TZ', '02733Z6', '02733ZZ', '0273446', '027344Z', '02734D6', '02734DZ', '02734T6', '02734TZ', '02734Z6', '02734ZZ', '02C03ZZ', '02C04ZZ', '02C13ZZ', '02C14ZZ', '02C23ZZ', '02C24ZZ', '02C33ZZ', '02C34ZZ', 'X2C0361', 'X2C1361'
**Coronary artery bypass graft procedure codes[1]**	33510', '33511', '33512', '33513', '33514', '33516', '33517', '33518', '33519', '33521', '33522', '33523', '33533', '33534', '33535', '33536'	0210093', '0210098', '0210099', '021009C', '021009F', '021009W', '02100A3', '02100A8', '02100A9', '02100AC', '02100AF', '02100AW', '02100J3', '02100J8', '02100J9', '02100JC', '02100JF', '02100JW', '02100K3', '02100K8', '02100K9', '02100KC', '02100KF', '02100KW', '02100Z3', '02100Z8', '02100Z9', '02100ZC', '02100ZF', '0210344', '02103D4', '0210444', '0210493', '0210498', '0210499', '021049C', '021049F', '021049W', '02104A3', '02104A8', '02104A9', '02104AC', '02104AF', '02104AW', '02104D4', '02104J3', '02104J8', '02104J9', '02104JC', '02104JF', '02104JW', '02104K3', '02104K8', '02104K9', '02104KC', '02104KF', '02104KW', '02104Z3', '02104Z8', '02104Z9', '02104ZC', '02104ZF', '0211093', '0211098', '0211099', '021109C', '021109F', '021109W', '02110A3', '02110A8', '02110A9', '02110AC', '02110AF', '02110AW', '02110J3', '02110J8', '02110J9', '02110JC', '02110JF', '02110JW', '02110K3', '02110K8', '02110K9', '02110KC', '02110KF', '02110KW', '02110Z3', '02110Z8', '02110Z9', '02110ZC', '02110ZF', '0211344', '02113D4', '0211444', '0211493', '0211498', '0211499', '021149C', '021149F', '021149W', '02114A3', '02114A8', '02114A9', '02114AC', '02114AF', '02114AW', '02114D4', '02114J3', '02114J8', '02114J9', '02114JC', '02114JF', '02114JW', '02114K3', '02114K8', '02114K9', '02114KC', '02114KF', '02114KW', '02114Z3', '02114Z8', '02114Z9', '02114ZC', '02114ZF',

	'0212093', '0212098', '0212099', '021209C', '021209F', '021209W', '02120A3', '02120A8', '02120A9', '02120AC', '02120AF', '02120AW', '02120J3', '02120J8', '02120J9', '02120JC', '02120JF', '02120JW', '02120K3', '02120K8', '02120K9', '02120KC', '02120KF', '02120KW', '02120Z3', '02120Z8', '02120Z9', '02120ZC', '02120ZF', '0212344', '02123D4', '0212444', '0212493', '0212498', '0212499', '021249C', '021249F', '021249W', '02124A3', '02124A8', '02124A9', '02124AC', '02124AF', '02124AW', '02124D4', '02124J3', '02124J8', '02124J9', '02124JC', '02124JF', '02124JW', '02124K3', '02124K8', '02124K9', '02124KC', '02124KF', '02124KW', '02124Z3', '02124Z8', '02124Z9', '02124ZC', '02124ZF', '0213093', '0213098', '0213099', '021309C', '021309F', '021309W', '02130A3', '02130A8', '02130A9', '02130AC', '02130AF', '02130AW', '02130J3', '02130J8', '02130J9', '02130JC', '02130JF', '02130JW', '02130K3', '02130K8', '02130K9', '02130KC', '02130KF', '02130KW', '02130Z3', '02130Z8', '02130Z9', '02130ZC', '02130ZF', '0213344', '02133D4', '0213444', '0213493', '0213498', '0213499', '021349C', '021349F', '021349W', '02134A3', '02134A8', '02134A9', '02134AC', '02134AF', '02134AW', '02134D4', '02134J3', '02134J8', '02134J9', '02134JC', '02134JF', '02134JW', '02134K3', '02134K8', '02134K9', '02134KC', '02134KF', '02134KW', '02134Z3', '02134Z8', '02134Z9', '02134ZC', '02134ZF'
**Percutaneous coronary intervention procedure codes[1] CPT codes**	92920', '92921', '92924', '92925', '92928', '92929', '92933', '92934', '92937', '92938', '92941', '92943', '92944', '92980', '92981', '92982', '92984', '92995', '92996', 'C9600', 'C9601', 'C9602', 'C9603', 'C9604', 'C9605', 'C9606', 'C9607', 'C9608', 'G0290', 'G0291'
**Coronary artery bypass graft procedure codes[1]**	33510', '33511', '33512', '33513', '33514', '33516', '33517', '33518', '33519', '33521', '33522', '33523', '33533', '33534', '33535', '33536'
**Stop codes identifying emergency visits**	130, 101, 1, 297, 131
**AHRQ-CCS = the Agency for Health Research and Quality Clinical Classifications Software[3]**	

<u>Supplemental Table S9:</u> Administrative codes (ICD-9 and ICD-10) used in definitions of baseline characteristics.

Table S9. Administrative codes (ICD-9 and ICD-10) used in definitions of baseline characteristics.		
**Baseline characteristic**	**ICD-9 Definition**	**ICD-10-definition**
Gender	Self-identified	
Race and ethnicity	Self-identified	
Family history of cardiovascular diseases	ICD-9-CM codes: V171, V1749, V174, V1741, and V173.	Z8249 Z8241 Z8674
Smoking	ICD-9-CM codes: 3051 and V1582	AHRQ-CCS category MBD024
Alcohol-related disorders	AHRQ-CCS category 660	AHRQ-CCS category MBD017
Substance-related disorders	AHRQ-CCS category 661	AHRQ-CCS category SYM009
Diabetes with ketoacidosis or uncontrolled diabetes	AHRQ-CCS category 3.3.1 and Selected codes from AHRQ-CCS category 3.3.7 (24920 24921 24930 24931 25020 25021 25022 25023 25030 25032 25033)	E0800 E0801 E0810 E0811 E0865 E0900 E0901 E0910 E0911 E0965 E1010 E1011 E1065 E1100 E1101 E1110 E1111 E1165 E1300 E1301 E1310 E1311 E1365
Diabetes with ophthalmic manifestations	24950 24951 25050 25051 25052 25053	E08311 E08319 E08321 E083211 E083212 E083213 E083219 E08329 E083291 E083292 E083293 E083299 E08331 E083311 E083312 E083313 E083319 E08339 E083391 E083392 E083393 E083399 E08341 E083411 E083412 E083413 E083419 E08349 E083491 E083492 E083493 E083499 E08351 E083511 E083512 E083513 E083519 E083521 E083522 E083523 E083529 E083531 E083532 E083533 E083539 E083541 E083542 E083543 E083549 E083551 E083552 E083553 E083559 E08359 E083591 E083592 E083593 E083599 E0836 E0837X1 E0837X2 E0837X3 E0837X9 E0839 E09311 E09319 E09321 E093211 E093212 E093213 E093219 E09329 E093291 E093292 E093293 E093299 E09331 E093311 E093312 E093313 E093319 E09339 E093391 E093392 E093393 E093399 E09341 E093411 E093412 E093413

	E093419 E09349 E093491 E093492<br>E093493 E093499 E09351 E093511<br>E093512 E093513 E093519 E093521<br>E093522 E093523 E093529 E093531<br>E093532 E093533 E093539 E093541<br>E093542 E093543 E093549 E093551<br>E093552 E093553 E093559 E09359<br>E093591 E093592 E093593 E093599<br>E0936 E0937X1 E0937X2 E0937X3<br>E0937X9 E0939 E10311 E10319 E10321<br>E103211 E103212 E103213 E103219<br>E10329 E103291 E103292 E103293<br>E103299 E10331 E103311 E103312<br>E103313 E103319 E10339 E103391<br>E103392 E103393 E103399 E10341<br>E103411 E103412 E103413 E103419<br>E10349 E103491 E103492 E103493<br>E103499 E10351 E103511 E103512<br>E103513 E103519 E103521 E103522<br>E103523 E103529 E103531 E103532<br>E103533 E103539 E103541 E103542<br>E103543 E103549 E103551 E103552<br>E103553 E103559 E10359 E103591<br>E103592 E103593 E103599 E1036<br>E1037X1 E1037X2 E1037X3 E1037X9<br>E1039 E11311 E11319 E11321 E113211<br>E113212 E113213 E113219 E11329<br>E113291 E113292 E113293 E113299<br>E11331 E113311 E113312 E113313<br>E113319 E11339 E113391 E113392<br>E113393 E113399 E11341 E113411<br>E113412 E113413 E113419 E11349<br>E113491 E113492 E113493 E113499<br>E11351 E113511 E113512 E113513<br>E113519 E113521 E113522 E113523<br>E113529 E113531 E113532 E113533<br>E113539 E113541 E113542 E113543<br>E113549 E113551 E113552 E113553<br>E113559 E11359 E113591 E113592<br>E113593 E113599 E1136 E1137X1<br>E1137X2 E1137X3 E1137X9 E1139 E13311<br>E13319 E13321 E133211 E133212<br>E133213 E133219 E13329 E133291<br>E133292 E133293 E133299 E13331

		E133311 E133312 E133313 E133319 E13339 E133391 E133392 E133393 E133399 E13341 E133411 E133412 E133413 E133419 E13349 E133491 E133492 E133493 E133499 E13351 E133511 E133512 E133513 E133519 E133521 E133522 E133523 E133529 E133531 E133532 E133533 E133539 E133541 E133542 E133543 E133549 E133551 E133552 E133553 E133559 E13359 E133591 E133592 E133593 E133599 E1336 E1337X1 E1337X2 E1337X3 E1337X9 E1339
Diabetes with renal manifestations	24940 24941 25040 25041 25042 25043	E0821 E0822 E0829 E0921 E0922 E0929 E1021 E1022 E1029 E1121 E1122 E1129 E1321 E1322 E1329
Diabetes with neurological manifestations	24960 24961 25060 25061 25062 25063	E0840 E0841 E0842 E0843 E0844 E0849 E08610 E0940 E0941 E0942 E0943 E0944 E0949 E09610 E1040 E1041 E1042 E1043 E1044 E1049 E10610 E1140 E1141 E1142 E1143 E1144 E1149 E11610 E1340 E1341 E1342 E1343 E1344 E1349 E13610
Diabetes with circulatory manifestations	AHRQ-CCS category 3.3.5	E0851 E0852 E0859 E0951 E0952 E0959 E1051 E1052 E1059 E1151 E1152 E1159 E1351 E1352 E1359
Diabetes with unspecified manifestations	AHRQ-CCS category 3.3.6	E08618 E08620 E08621 E08622 E08628 E08630 E08638 E0869 E088 E09618 E09620 E09621 E09622 E09628 E09630 E09638 E0969 E098 E10618 E10620 E10621 E10622 E10628 E10630 E10638 E1069 E108 E11618 E11620 E11621 E11622 E11628 E11630 E11638 E1169 E118 E13618 E13620 E13621 E13622 E13628 E13630 E13638 E1369 E138
Diabetes with hypoglycemia	2510 2511 2512 2703 7750 7756 9623	E08641 E08649 E09641 E09649 E10641 E10649 E11641 E11649 E13641 E13649 E15 E160 E161 E162 T383X1A T383X1D T383X1S T383X2A T383X2D T383X2S T383X3A T383X3D T383X3S T383X4A T383X4D T383X4S T383X5A T383X5D T383X5S
Hemiplegia/quadreplegia	CCI using Deyo method	CCI using Beyrer validated codes
Dementia	CCI using Deyo method	CCI using Beyrer validated codes

Liver disease-mild	CCI using Deyo method	CCI using Beyrer validated codes
Liver disease-severe	CCI using Deyo method	CCI using Beyrer validated codes
Malignancy other than skin cancer	CCI using Deyo method	CCI using Beyrer validated codes
Metastatic neoplasm	CCI using Deyo method	CCI using Beyrer validated codes
Acquired Immunodeficiency Syndrome	CCI using Deyo method	CCI using Beyrer validated codes
Hypertension	AHRQ-CCS category 98	AHRQ-CCS category CIR007
Hypertension with complications and secondary hypertension	AHRQ-CCS category 99	AHRQ-CCS category CIR008
Diabetes	AHRQ-CCS category 49	AHRQ-CCS category END002
Diabetes with complication	AHRQ-CCS category 50	AHRQ-CCS category END003
Rheumatoid arthritis and related disease; Systemic lupus erythematosus and connective tissue disorders	AHRQ-CCS category 202 & 210	AHRQ-CCS category MUS003
schizophrenia and other psychotic disorders	AHRQ-CCS category 659	AHRQ-CCS category MBD001
suicide and intentional self-inflicted injury	AHRQ-CCS category 662	AHRQ-CCS category MBD027
Anemia	VAFI codes	VAFI codes
Atrial fibrillation	VAFI codes	VAFI codes
Thyroid disease	VAFI codes	VAFI codes
Gait abnormality	VAFI codes	VAFI codes
Arthritis	VAFI codes	VAFI codes
Falls	VAFI codes	VAFI codes
Incontinence	VAFI codes	VAFI codes
Muscle wasting	VAFI codes	VAFI codes
Osteoporosis	VAFI codes	VAFI codes
Parkinsonism	VAFI codes	VAFI codes
Peripheral neuropathy	VAFI codes	VAFI codes
Impaired vision	VAFI codes	VAFI codes
Weight loss	VAFI codes	VAFI codes
Anxiety	VAFI codes	VAFI codes
Depression	VAFI codes	VAFI codes
Chronic pain	VAFI codes	VAFI codes
Failure to thrive	VAFI codes	VAFI codes
Fatigue	VAFI codes	VAFI codes
Congestive heart failure	VAFI codes	VAFI codes
Coronary artery disease	VAFI codes	VAFI codes

Obesity	VAFI codes	VAFI codes

AHRQ-CCS = the Agency for Health Research and Quality Clinical Classifications Software[3]

*CCI using Deyo et al method in calculating the Charlson comorbidity index. [4]

CCI using validated set of ICD-10 codes by Beyrer et al[5]

VAFI codes denote VA-Frailty index using Orkaby et al method, which was validated in VA population. [6]

Supplemental Table S10: LOINC codes used to identify laboratory tests

**LOINC codes used to identify laboratory tests**	
Laboratory test name	LOINC codes
Hemoglobin A1C (Glycohemoglobin)	17855-8, 17856-6, 4548-4, 4549-2
LDL-Cholesterol (both calculated and direct)	13457-7, 18262-6, 2089-1, 49132-4
HDL-Cholesterol	2085-9
Total Cholesterol	2093-3
Serum Creatinine	2160-0

Table S11: Handling of missing values at baseline

**Handling of missing lab values at baseline**				
	**Total**	**Missing**	**Replaced by mean**	**Still missing**
DDP4 basic cohort				
A1C	37,999	151	129	22
Glucose	37,999	65	61	4
Creatinine	37,999	60	60	0
LDL_Cholest	37,999	250	188	62
HDL_Cholest	37,999	241	185	56
T_Cholesterol	37,999	269	208	61
GLP1 basic cohort				
A1C	27,146	35	22	13
Glucose	27,146	12	12	0
Creatinine	27,146	5	5	0
LDL_Cholest	27,146	111	75	36
HDL_Cholest	27,146	96	64	32
T_Cholesterol	27,146	93	61	32
**Handling missing vitals at baseline**				
	**Total**	**Missing**	**Replaced by mean**	**Still missing**
DDP4 basic cohort				
Weight	37,999	87	69	18
Systolic/ Diastolic BP	37,999	76	61	15
GLP1 basic cohort				
Weight	27,146	39	32	7
Systolic/ Diastolic BP	27,146	30	23	7

1. Fishman E, Barron J, Dinh J, et al. Validation of a claims-based algorithm identifying eligible study subjects in the ADAPTABLE pragmatic clinical trial. *Contemp Clin Trials Commun*. Dec 2018;12:154-160. doi:10.1016/j.conctc.2018.11.001
2. Kivimäki M, Batty GD, Singh-Manoux A, Britton A, Brunner EJ, Shipley MJ. Validity of Cardiovascular Disease Event Ascertainment Using Linkage to UK Hospital Records. *Epidemiology*. 2017;28(5)
3. Elixhauser A, Steiner C, Palmer L. Clinical Classifications Software (CCS) for ICD-9-CM. *Databases and Related Tools from the Healthcare Cost and Utilization Project (HCUP)* U.S. Agency for Healthcare Research and Quality; 2012:Appendix A. Accessed 01/01/2012. http://www.hcup-us.ahrq.gov/toolssoftware/ccs/ccs.jsp (last accessed March 16, 2015)
4. Deyo RA, Cherkin DC, Ciol MA. Adapting a clinical comorbidity index for use with ICD-9-CM administrative databases. *Journal of clinical epidemiology*. Jun 1992;45(6):613-9. doi:0895-4356(92)90133-8 [pii]
5. Beyrer J, Manjelievskaia J, Bonafede M, et al. Validation of an International Classification of Disease, 10th revision coding adaptation for the Charlson Comorbidity Index in United States healthcare claims data. *Pharmacoepidemiol Drug Saf*. May 2021;30(5):582-593. doi:10.1002/pds.5204
6. Orkaby AR, Nussbaum L, Ho YL, et al. The Burden of Frailty Among U.S. Veterans and Its Association With Mortality, 2002-2012. *J Gerontol A Biol Sci Med Sci*. Jul 12 2019;74(8):1257-1264. doi:10.1093/gerona/gly232
